# Investigating the dispersal of antibiotic resistance associated genes from manure application to soil and drainage waters in simulated agricultural farmland systems

**DOI:** 10.1371/journal.pone.0222470

**Published:** 2019-09-17

**Authors:** Schuyler D. Smith, Phillip Colgan, Fan Yang, Elizabeth L. Rieke, Michelle L. Soupir, Thomas B. Moorman, Heather K. Allen, Adina Howe

**Affiliations:** 1 Bioinformatics and Computational Biology Department, Iowa State University, Ames, Iowa, United States of America; 2 Department of Agricultural and Biosystems Engineering, Iowa State University, Ames, Iowa, United States of America; 3 United States Department of Agriculture, Agricultural Research Service, Ames, Iowa, United States of America; USDA-ARS Salinity Laboratory, UNITED STATES

## Abstract

Manure from animals that have been treated with antibiotics is often used to fertilize agricultural soils and its application has previously been shown to enrich for genes associated with antibiotic resistance in agroecosystems. To investigate the magnitude of this effect, we designed a column experiment simulating manure-treated agricultural soil that utilizes artificial subsurface drainage to determine the duration and extent which this type of manure fertilization impacts the set of genes associated with antibiotic resistance in drainage water. We classified ARGs in manure-treated drainage effluent water by its source of origin. Overall, we found that 61% and 7% of the total abundance of ARGs found in drainage water samples could be attributed to manure enrichment and manure addition, respectively. Among these ARGs, we identified 75 genes unique to manure that persisted in both soil and drainage water throughout a drainage season typical of the Upper Midwestern United States. While most of these genes gradually decreased in abundance over time, the IS6100-associated *tet(33)* gene accrued. These results demonstrate the influence of manure applications on the composition of the resistome observed in agricultural drainage water and highlight the importance of anthropogenic ARGs in the environment.

## Introduction

Antimicrobial resistance (AMR) is a global threat [1]. Infections associated with AMR have resulted in approximately 700,000 deaths each year worldwide [2]. With global antibiotic consumption expected to increase by 67% by 2030 [3], that number is expected to rise to near 10 million by 2050 [2]. In response to this problem, regulatory organizations around the world have called for the development of strategies and regulations for the responsible management of anthropogenic antibiotic-resistant bacteria (ARB) and antibiotic resistance gene (ARG) pollution [4].

Bacterial antibiotic resistance has been a known clinical issue since the first use of sulfonamides and penicillin in the late 1930’s and early 1940’s for treating human patients with bacterial infections [5]. Since that time, antibiotic usage has expanded into animal agriculture in quantities that surpassed the amount of antibiotics used in human clinical settings [3]. Sub-therapeutic doses of antibiotics have been used to improve feed efficiency and encourage growth promotion in livestock [6]. The 2017 FDA ruling deemed it unlawful to use human applicable antibiotics for the purposes of animal growth promotion in the United States, but there has been continued usage of other antibiotics in livestock production to protect animal health.

The use of antibiotics in animal production has been shown to increase the abundance of ARBs and ARGs within the gut microbial communities and feces of these animals [7–9]. Studies have shown that fertilization of agricultural soils with manure collected from these antibiotic-treated animals introduced novel ARGs into the associated soil microbial community, as well as enrichment of ARGs that were naturally present [8,10–17].

Observed enrichment of ARBs and ARGs in soils surrounding animal production can be further compounded by the presence of artificial subsurface drainage systems underneath agricultural soils. Farmland in the upper midwestern United States is often modified with artificial subsurface drainage systems to protect crops from flooding by redirecting excess rain-water to nearby rivers and streams. In the state of Iowa, an estimated 25 to 35% of cropland utilizes subsurface drainage systems, representing approximately 8 million acres [16]. The ability to transport elements from farmland soil via the waterways has been well studied for macronutrients such as nitrogen [18–20].These nutrients are applied to soil as fertilizer and have been observed to move into the water systems in such vast quantities that they have negatively affect water quality [21]. Previous field studies have also compared the movement of pathogens (Enterococcus), antibiotic-resistant bacteria, and antibiotic resistant genes (*ermB* and *ermF*) conferring resistance to macrolide antibiotics after injection of swine manure to soil. In soil receiving the manure injection, copies of *ermB*, *ermF*, and tylosin-resistant *Enterococcus* increased substantially as these were present in the swine manure and concentrations of these genes remained elevated in soil over the levels found in the soil without manure application over the winter after fall application [22]. Concentrations of *Enterococcus* and *erm* genes were lower in tile drainage water than in soil, but water draining from manure-treated soil contained more *ermB* and *ermF* than drainage from soil s without manure in years with more than average precipitation [23].

The potential of ARBs and ARGs to spread throughout the environment has led to their classification as emerging contaminants [24]. In order to determine the impact our farming practices have on the suite of ARGs in the environment, or the resistome, we must differentiate between anthropogenic ARG contamination and background levels of ARGs that naturally occur in the farmland environment [17,25–29]. This ability to monitor anthropogenic ARGs will be central to developing strategies to inform regulations regarding responsible management of ARG pollution. To gain evidence of the effect of manure-associated ARGs moving through soil and into drainage water, we designed a soil column experiment modelling manure-treated cropland that uses artificial subsurface drainage. The diversity and abundance of ARGs in both the soil and drainage water were monitored following six intermediate rainfall simulations spanning a 108-day drainage season. The objective of this experiment was to determine the duration and extent to which manure fertilization of these soils impacts the environmental resistomes, as well as characterize the antibiotic resistance classes that are most associated with genes originating from manure.

## Methods

### Soil columns

Soil column construction, experimental design and sampling procedures were previously described in detail by [30]. This study utilized soil and effluent DNA extracts resulting from manured and non-manured soil columns which were maintained under chisel plow management. Briefly, soil columns were extracted from the Iowa State University Northeast Research and Demonstration Farm, near Nashua, IA, United States (43.0°N, 92.5°W). These soils either have a known manure treatment history, with the last application occurring two years prior to soil collection or no manure treatment over the past 30 years. Soil column depths ranged from 46–51 cm from the soil surface. The depth of column was chosen to mimic typical depths to artificial tile drainage. The experiment was performed at Iowa State University, under typical building temperature conditions. Manure and non-manured sets of soil columns were destructively sampled prior to manure application and 24, 59, and 108 days following manure application. The top 15 cm of soil from each column was extracted, homogenized by hand, and sub-sampled for DNA extraction. Six simulated rainfall events (each event represented by 1 L deionized water) were conducted over the course of the 108 day experiment. Effluent collected 10 and 24 days after manure application was derived from soil columns destructed on Day 24. Similarly, effluent collected 38 and 59 days after manure application was derived from columns destructed on Day 59 and effluent collected 80 and 108 days after manure application was derived from columns destructed 108 days after manure application. Leachate was collected in 1 L sterile Nalgene bottles located on racks below the columns.

Manure was obtained from a swine finishing facility that supplemented antibiotics in feed. These antibiotics included both macrolide and tetracycline antibiotics (personal communication), in accordance with standard swine production commercial practices. Manure was tested for the presence of antibiotics by the Water Sciences Laboratory at the University of Nebraska, as previously described [28, 29].

### DNA sequencing

DNA was extracted from soil and manure as control references before the experiment was conducted. Eighteen of the soil columns were fertilized with manure treatment, and the other half were used as controls receiving no manure treatment; all were subjected to the simulated rainfall. The 6 rainfall events were performed on days 10, 24, 38, 59, 80, and 108 after manure treatment (day 0). After each rainfall, drainage water was collected from all columns for DNA extraction. Manure-treated soil columns were destructively sampled on days 24, 59, and 108. DNA was extracted as previously described in [31]. For every DNA extraction in this study, 4 replicates were taken, totaling 56 samples collected.

Shotgun metagenome sequencing was performed on the 56 samples (S1 Table). Sequencing was performed at the Iowa State University DNA Facility using an Illumina HiSeq 3000 platform with 150 bp paired-end sequencing. Metagenome libraries ranged from 8.4 to 50.1 Mbp. Trimmomatic (version 0.33) [32] was used for Nextera PE adapter removal and sequence quality trimming with removal of leading and trailing low quality or N bases below quality 3, trimming when the average quality per base drops below 15 in any 4-base sliding window, and removal of reads less than 36 bp in length. The quality-trimmed sequencing reads were compared to known genes in the Comprehensive Antibiotic Resistance Database (CARD, version 2.0.1) [33] using BLAST (version 2.4.0) [34]. For a sequence to be considered matched to a known CARD gene, both read pairs from the metagenome were required to have a minimum alignment E-value of 1e-5. If multiple genes matched for the same read pair, the alignment with the best score was selected. To estimate the abundance of a gene, the total number of observed reads for that gene was normalized by the number of *recA* gene sequences (Fungene *recA* gene database, version 9.6, [35]) observed in each corresponding metagenome, giving an estimate of the per-cell abundance.

### Data analysis

Analysis was performed within the R software environment using the phyloseq data-standard [36] and analyzed using the phylosmith package [37].

#### Identify source derived ARGs

**Fig 1:**
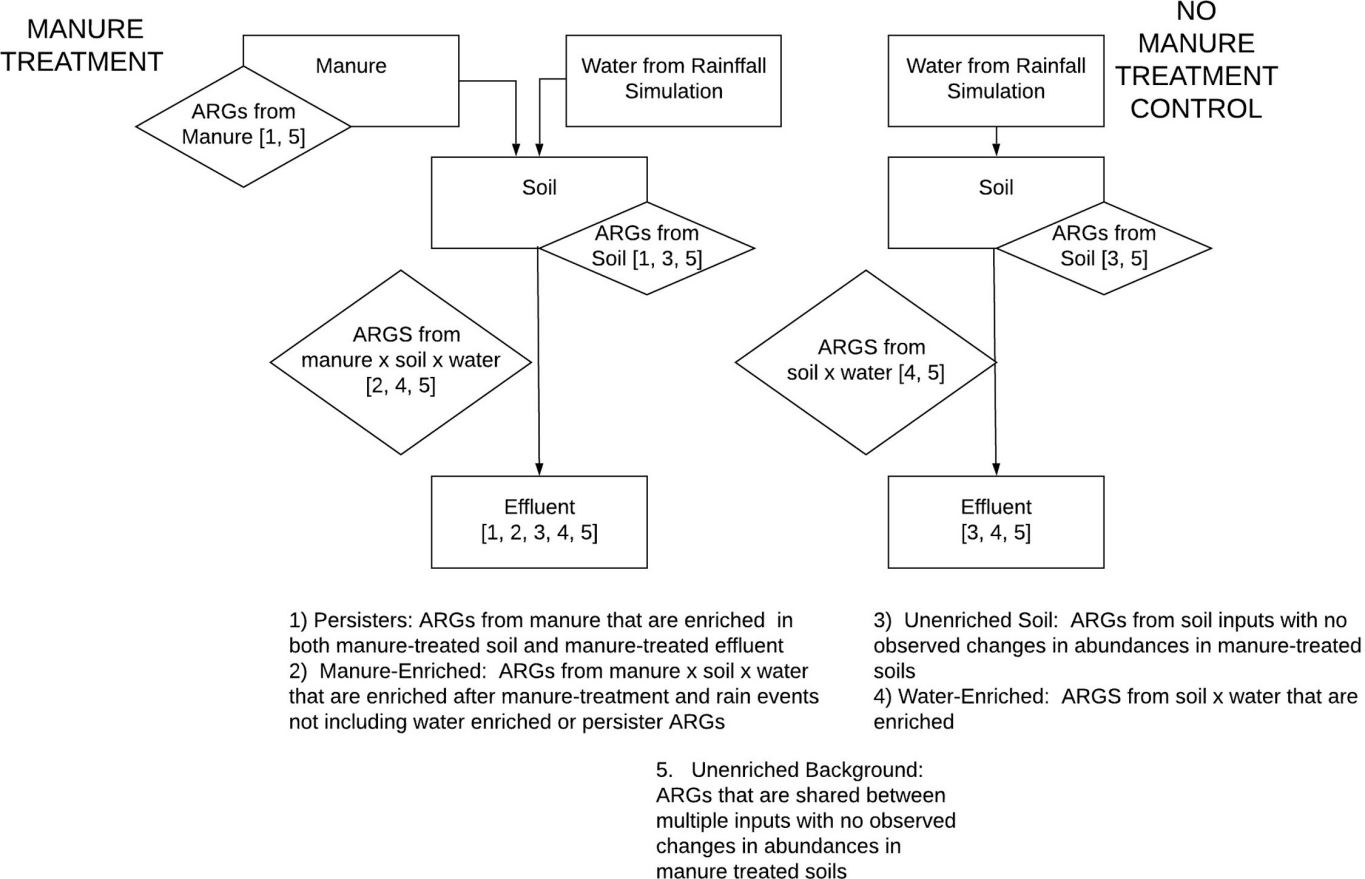
Classification of potential sources of ARGs in effluent from manure-treated and untreated soil columns. ARGs were categorized based on presence in one or multiple sources and observation of enrichment throughout the study. Potential ARGs from each source are shown in brackets.

The presence of ARGs was compared between control samples (soil and manure) (Fig 1). This comparison determined which genes were shared between the two sources and which were uniquely derived, or sourced, from each. Manure-derived genes that were found to be present in the manure-treated columns after rainfall events were labeled “persisters”, as they persisted in the environment after introduction. Genes that were common between the manure and soil were considered to have a potential to be naturally occurring, along with the soil-derived genes.

#### Classify sources of enrichment

To identify how genes that are naturally present in the soil were affected by the manure treatment and rainfall, we examined the total abundance of ARGs in each treatment for each sample. Any gene seen in a higher abundance relative to the control soil in the effluent-control samples was labeled as “water-enriched”. Any genes that were not water-enriched that were seen to increase in abundance in the manure-treated columns relative to the control-soil were labeled as “manure-enriched”. These genes could include the genes that were observed to be present in both the soil and manure control samples.

#### Sample variance

The abundance of each ARG for the technical replicates for each sample were compared with the vegan::adonis() function in R [38]. This function is an implementation of a statistical ANOVA applied to distance matrices. This test allowed us to determine if replicates should be treated as independent or could be analyzed together in subsequent tests. To quantify the variation between samples we used pair-wise Mann-Whitney testing, stats::wilcox.test(). The t-Distributed Stochastic Neighbor Embedding (t-SNE) is a machine-learning method that uses distance matrices for ordination of datasets. This was applied to the dataset here using Bray-Curtis distance measurement to visualize how the variation between samples could be plotted in space. Along with the dimensionality reduction, 95% confidence intervals of the variance were calculated as ellipses to be displayed with each sample type.

## Results

Antibiotics detected in manure used in the column experiments included high concentrations of tetracyline, 4890.1 ng/g dry weight, and oxytetracycline, 748.7 ng/g dry weight. Additionally, 230.6 ng/g dry wt of ractopamine, 18.8 ng/g dry wt of tiamulin, 15.9 ng/g dry weight tylosin, and 1.3 ng/g dry wt of monensin were observed.

Shotgun sequencing produced on average 30 million read sequences per sample, with a standard deviation of 13 million. The mean percentage of reads associated with a CARD ARG in control soil and manure samples were 0.002% and 0.198%, respectively. Manure-treated soil and effluent metagenomes were associated with a mean of 0.028% and 0.009% ARG reads, and untreated effluent metagenomes were associated with a mean of 0.005% ARG reads.

No significant differences were found between replicate sample resistomes (p = 1). Significant differences were observed between the resistome of manure and soil (p < 0.05) and soil and manure-treated soil (p < 0.05), based on pair-wise statistical analysis with Mann-Whitney testing of the variance.

**Fig 2:**
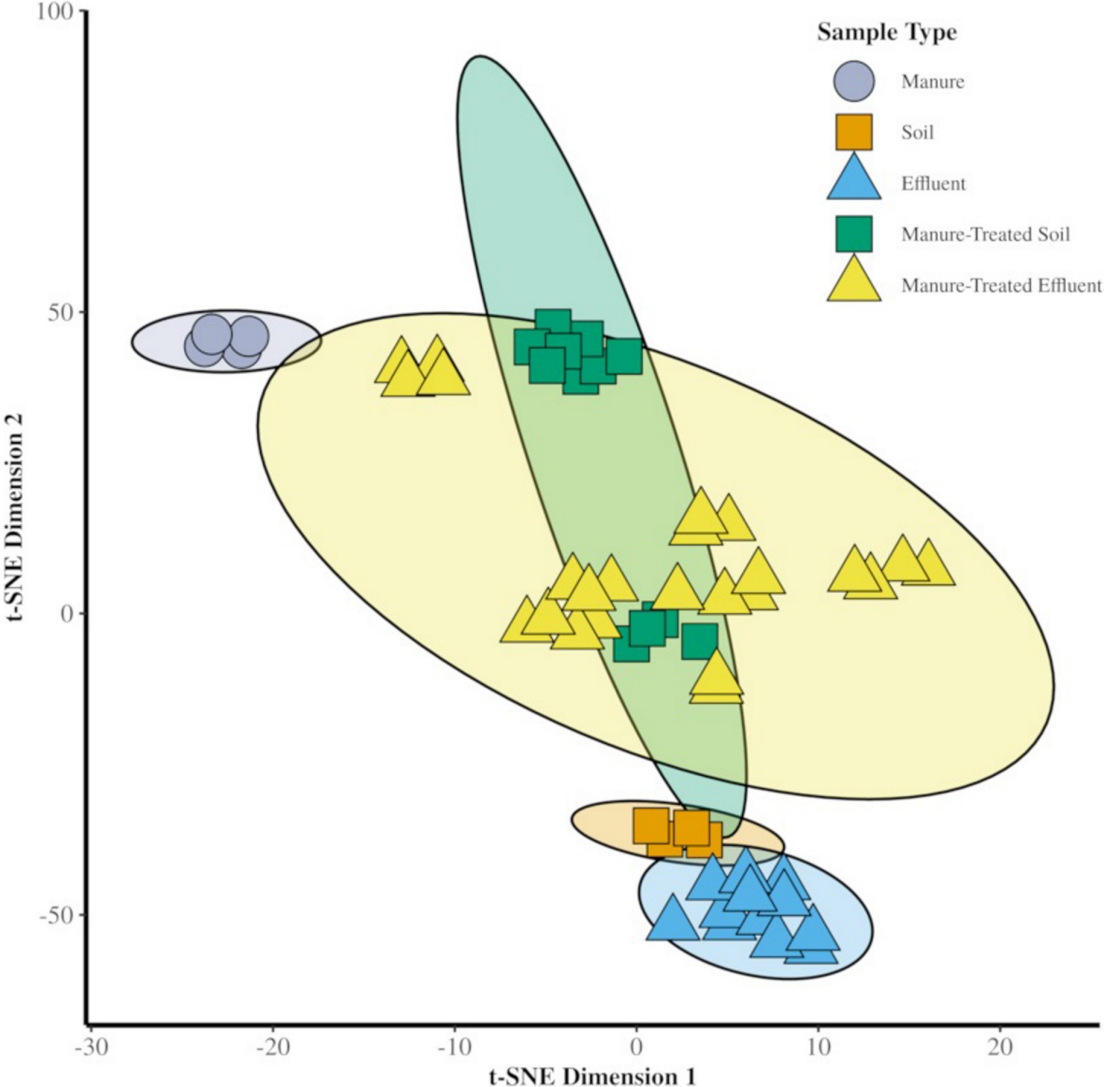
t-SNE dimension reduction using the Bray-Curtis distance of the 56 samples, based on read-sequence relative abundance. Different shapes represent different sample sources (e.g., both control soil and manure-treated soil samples are squares). The ellipse represents 95% confidence intervals of the variance for each sample type.

Plotting the t-SNE ordination (Fig 2), separation of untreated soil and manure-treated soil were seen along the two axes. Sub-clusters could be identified that corresponded with groupings of the 4 replicate samples representing the same sampling days, indicating the effect of time on the resistome composition that was not found to be statistically significant.

**Fig 3:**
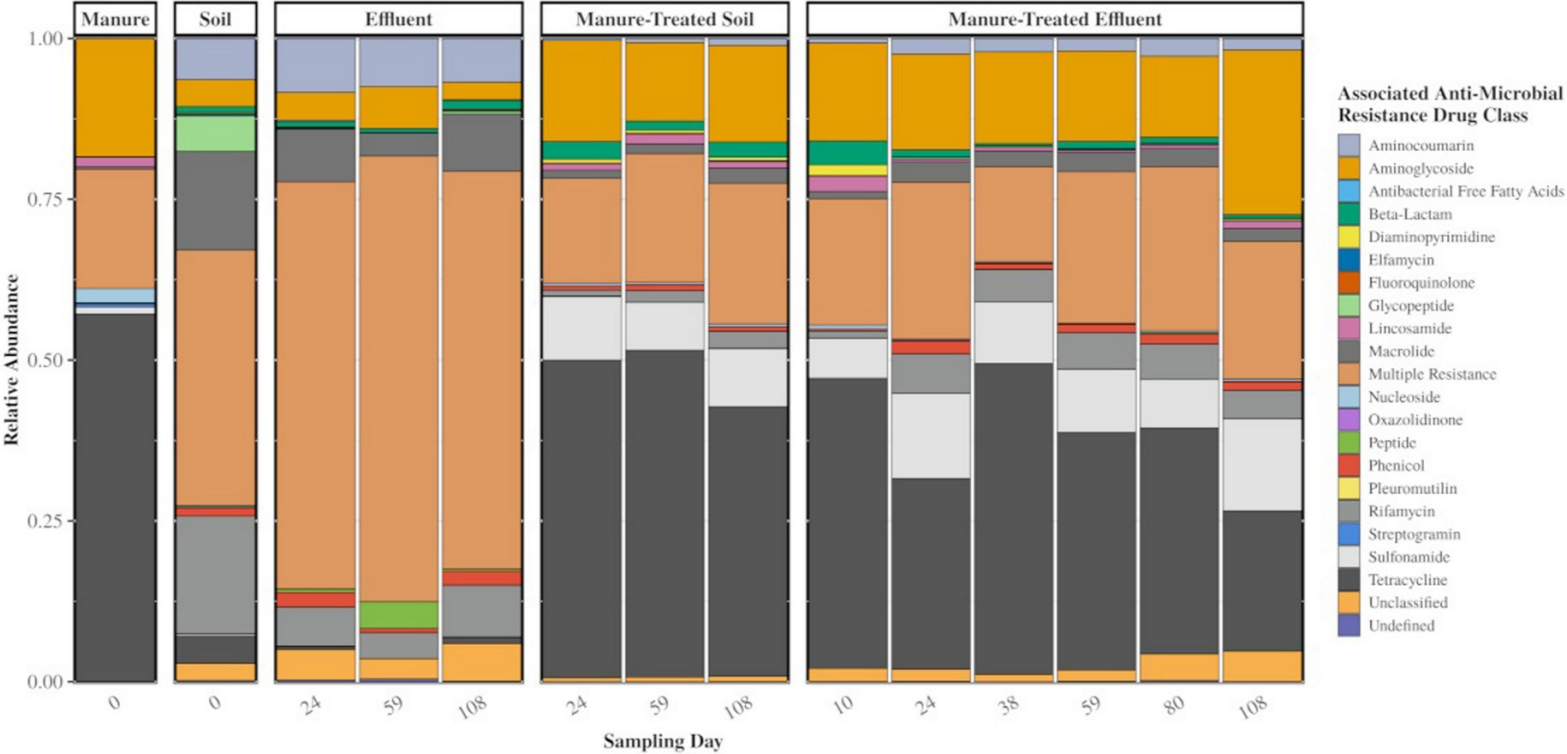
Relative abundance of ARGs (averaged for four replicate samples at each time point) categorized by associations with antibiotic resistance class in the CARD database. Samples are displayed in groupings by sample type. “Multiple Resistance” for ARG classification refers to any gene that has been annotated as associated with more than a single antibiotic class.

In the metagenomes from the study, 320 unique genes associated with antibiotic resistance were identified (S2 Table). Of these, 173 were found in the control manure samples and 176 in control soil samples; 85 ARGs were observed in the resistome shared between manure and soil samples. By read count, the manure resistome was observed to be 57% composed of ARGs associated with resistance to tetracycline antibiotics, 19% of associated to multiple classes of antibiotics, and 18% of associated to aminoglycoside antibiotics. In contrast, the majority of the soil resistome was 63% composed of genes associated with resistance to multiple classes of antibiotics (Fig 3).

**Fig 4:**
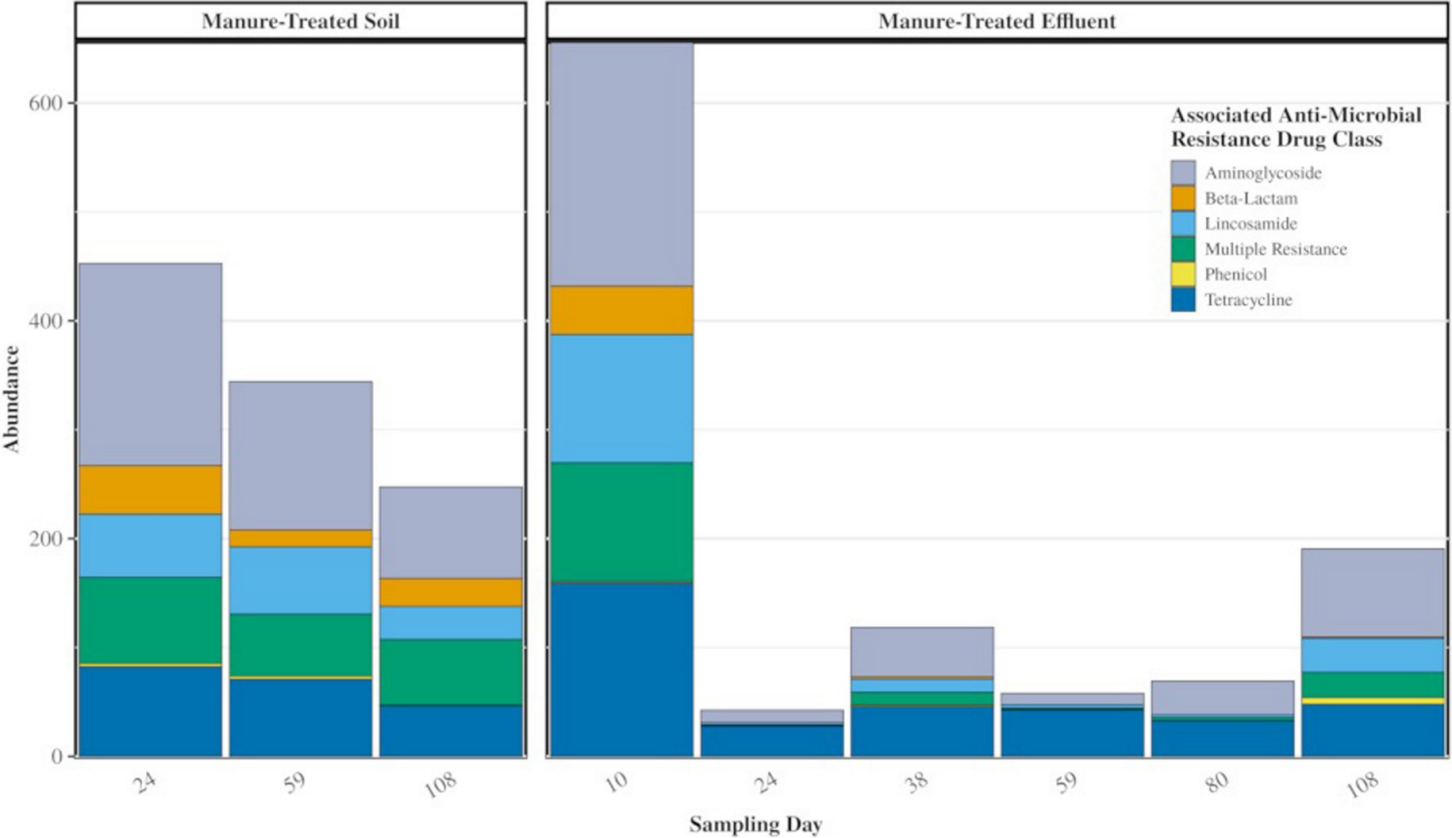
Abundance of persister ARGs observed in manure-treated soil and manure-treated effluent samples after each rain event. Abundance is averaged for 4 replicate samples at each time point and normalized by number of *recA* genes present in each sample. “Multiple Resistance” for ARG classification refers to any gene that has been annotated as associated with more than a single antibiotic class.

Of the 88 ARGs unique to the manure resistome, 75 were detected in manure-treated soil samples, and 69 were detected in manure-treated effluent samples. These persisters were comprised mostly of genes associated with resistance to aminoglycoside (49%) and tetracycline antibiotics (22%) (Fig 4).

**Fig 5:**
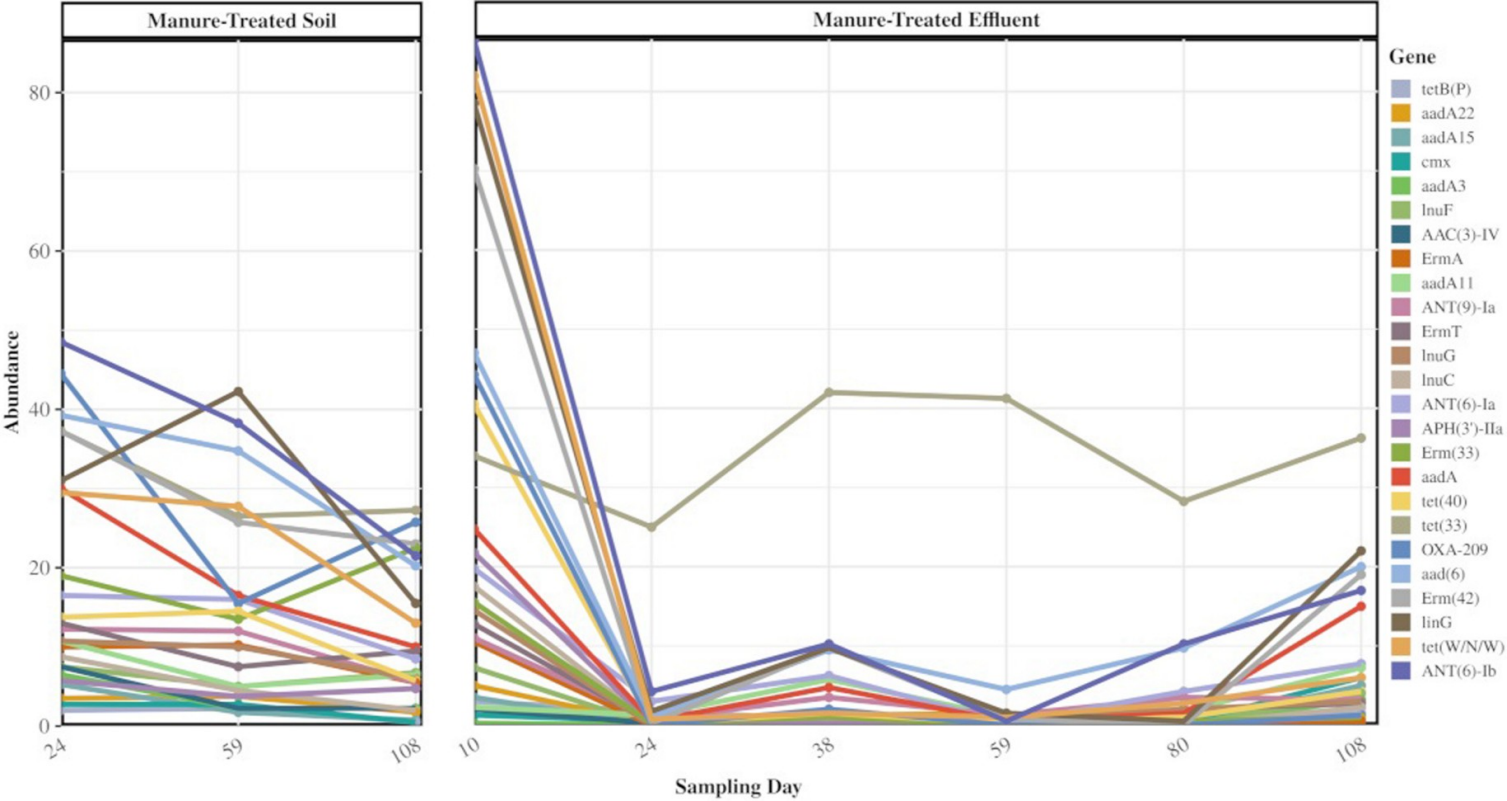
Abundance of 25 persister ARGs observed in manure-treated soil and manure-treated effluent samples in at least half of all samples after each rain event. Abundance is averaged for 4 replicate samples at each time point and normalized by number of *recA* genes present in each sample. *tet(33)* is highlighted, showing unique presence compared to the other persisters.

The persisters comprised 7% of the abundance of genes detected in the manure-treated effluent (Table 1). Twenty-five of these genes exhibited strong persistence, appearing in at least half of all manure-treated samples across all time points (Figs 5 and S1). Most of these persisters decreased in abundance over time in both soil and effluent, with the exception of sequences associated with *tet(33)*, which increased in abundance over time in the manure-treated effluent.

**Table 1:** The percentage and origin of ARGs in manure-treated effluent. ARG percentage represents the estimated percentage of abundance of observed ARGs.

Classification	ARG Percentage
Manure Enriched	0.612
Water Enriched	0.308
Manure Persister	0.071
Soil	0.003
Background	0.006

**Fig 6:**
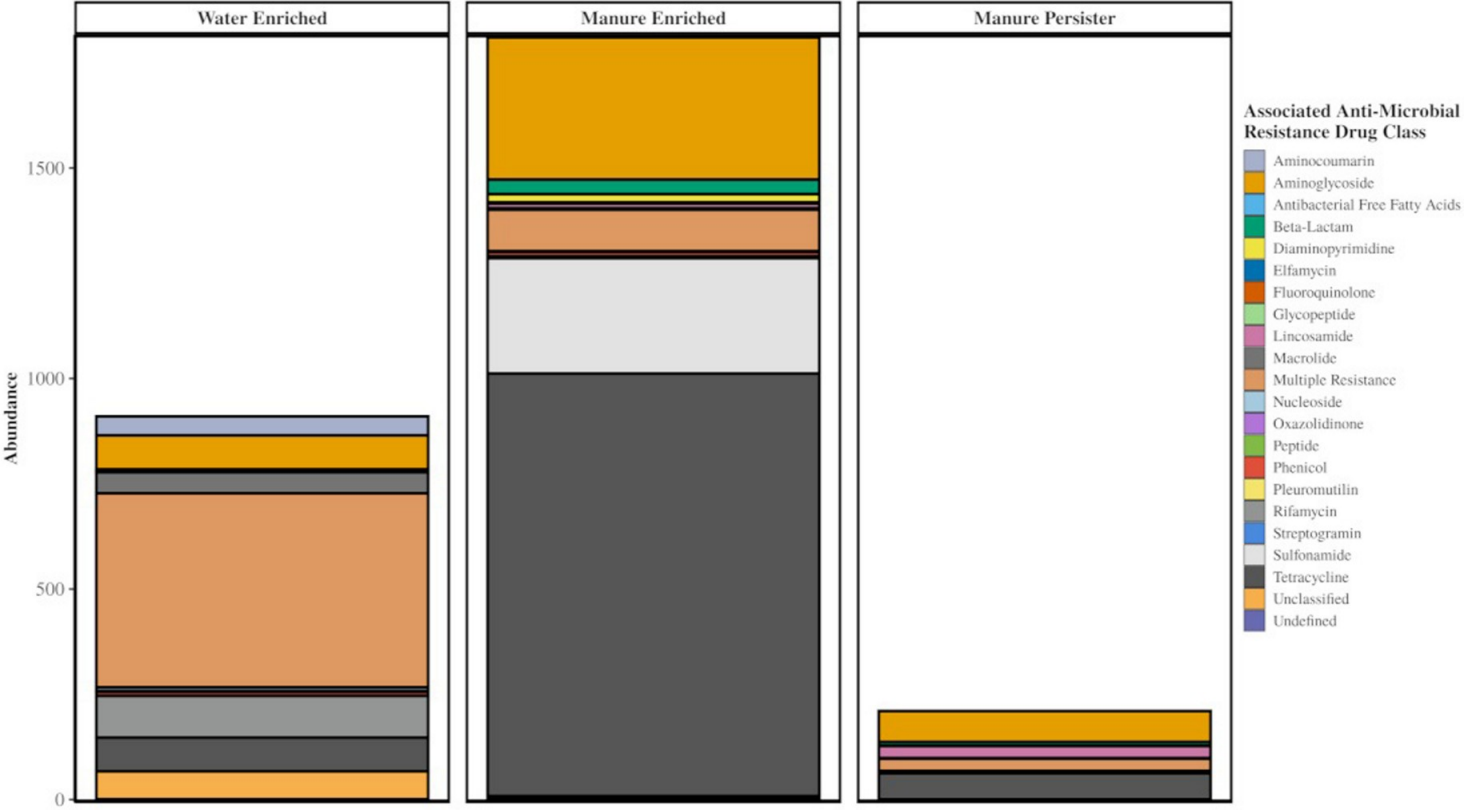
Abundance of ARGs observed in manure-treated effluent samples, summed across all sampling days. Abundance normalized by number of *recA* genes present. “Multiple Resistance” for ARG classification refers to any gene that has been annotated as associated with more than a single antibiotic class.

From the 176 observed ARGs in the untreated soil, 98 were water-enriched, which accounted for 30% of the abundance in the manure-treated effluent. There were also 63 manure-enriched ARGs in soils, accounting for 60% of the manure-treated effluent ARG abundance (Fig 6).

## Discussion

The manure used in this study was collected from swine that had received macrolide and tetracycline antibiotics in feed, which corresponded to the high abundance of tetracycline resistance genes in metagenomes of that manure (57%) that we observed. We found that these manures are associated with more abundant and contrasting resistome to the soils.

Similar to a previous study that characterized the ARG composition of pristine soils [17], the mechanism of action for the majority of the ARGs observed in untreated soil was related to mainly efflux of antibiotics from the cell via multi-drug efflux pumps. These types of multi-drug efflux pumps generally remove toxic metabolites [39–41] in addition to conferring antibiotic resistance. Thus, in soils, it is possible that these genes may be co-selected for by factors unrelated to antibiotics. Phylogenetic inference of the ARG sequences detected in the soil indicates that most observed genes are likely chromosomally located in typical soil bacteria. In comparison, the majority of manure-derived ARGs found were associated with various mobile genetic elements (MGEs) in known food- and water-borne pathogens, including *Campylobacter jejuni*, *Chlostridium perfringens*, and *Chlostridioides difficile*. These MGEs include plasmids, transposons, and integrons that may enhance the risk for mobility and transfer of associated ARGs between members of a manure-amended soil microbial community. The most abundant persisting ARG sequence found in manure-applied soil was homologous to an aminoglycoside resistance gene, ANT(6)-Ib, found on a transferrable pathogenicity island in *Campylobacter fetus* [42]. Additionally, one of the most abundant ARG sequences found in manure-applied soil shared homology with the gene *tet(33)*. This gene was previously found on a multi-drug resistant conjugative plasmid, pTET3, that harbors additional mobile genetic elements such as IS6100 insertion sequences, an integrase and integron cassettes [43].

The ordination of the resistomes for each sample highlights the effect of the manure application to the soil and drainage water effluent (Fig 2). The manure and control soil samples, including the control effluent that consists of only ARGs derived from the soil, reside on opposing quadrants of the graph, indicative of their contrasting resistomes. The manure-treated samples, both soil and effluent, can be seen between the control samples, evidence to the influence of manure treatment in shifting the native resistomes.

We observed an increase in relative abundance of ARGs in the soil directly after manure application that remained detectable but gradually attenuated over time (Fig 3), which is in agreement with similar studies investigating the temporal impact of manure application on the resistome of agricultural soil [44–46]. The three most indicative AMR classes of the manure resistome are the tetracyclines, aminoglycoside, and sulfonamides. The tetracycline and aminoglycoside ARGs are observed in the control soils, but in low relative abundance, while the sulfonamide ARGs are not present at all. After manure application to the soil, the tetracycline and aminoglycoside AMR classes became the majority represented groups in the resistome, and sulfonamides have a noticeable presence. Over time there is a slow shift back towards the control soil resistome, with the increasing presence of phenicol, beta-lactam, aminocoumarin, and unclassified AMR classes.

Although the observed shift back to the native soil resistome could be the result of the dominating presence of the soil ARGs, it is also likely that the introduced ARGs from manure are being flushed out of the system, as evidenced by the presence of these ARGs in effluent water samples. We also observe that ARGs persist in the environment at contrasting capacities. While most ARGs from manure seen in effluent tended to decrease in abundance in effluent over time, a total of 25 ARGs of the original 88 persisters remained in effluent samples throughout the study and some were also observed to be enriched in abundance. Sequences matching the *tet(33)* were associated with a notable trend in manure-treated effluent. The relative abundance of sequences associated with *tet(33)* in manure-treated soil followed the same general trend of decreasing presence, but increased after day 59, becoming the most abundant by day 108. In the treated effluent, the relative abundance of sequences associated with *tet(33)* was initially high but was not observed to dissipate between days 10 and 24 like many of the more abundant persisters. This gene is homologous to a sequence found on several plasmids that is flanked by IS6100 insertion elements, increasing its potential for mobility through transposition. IS6100 insertion elements were previously found to co-occur with clusters of genes conferring multiple antibiotic resistance in swine manure and also with class 1 integrase genes [47]. Our observations of sequences associated with *tet(33)* are also consistent with the measurable concentrations of tetracycline antibiotics in the manure and its previous associations with multiple MGEs. Its persistence and enrichment in manure treated effluent make this gene and its associated with manure impact key targets for future studies.

The largest proportion of ARGs found in the manure-treated effluent could be detected, though in low abundances, in untreated soils, but were enriched after manure application and rain events. The enrichment of these genes could be explained by increased water content an influx of nutrients that enhance microbial growth [10], or by direct addition from the manure microbiome, as 85 ARGs were shared between them.

The extent of the impact of manure fertilization is determined by both the manure-enriched and persister ARGs. These genes comprised 80% of the abundance of ARGs detected in manure and 68% of that observed in manure-treated effluent. These same manure-enriched genes represented only 1% of the abundance of ARGs in effluent of untreated soil. The high proportion of ARGs from manure treatment highlights the impact manure treatment had on the resistome in effluent waters in our study.

Our study provides evidence that manure-derived genes have a strong potential to not only persist in agricultural soil but also in water in areas that utilize artificial subsurface drainage systems. The results demonstrate the need to better understand the diversity of ARGs and their varying mechanisms of persistence and dissemination and the long-term effects they have on the environment. Because of the increasing global challenge of antibiotic resistance, we have been increasingly aware of the need to monitor the fate and transport of ARGs and ARBs in the environment, and our study provides further rationale to support these efforts. Importantly, for this study, shotgun metagenomic sequencing methods were used to detect and evaluate a broad diversity of ARGs and identify their potential associating ARBs. It is notable, however, that ARGs comprised only <0.01% of soil and water metagenomes. Consequently, metagenomic sequencing for large scale monitoring efforts would require extensive sequencing and high costs. This study highlighted genes that are associated with swine manure into soil and water that can be detected with targeted approaches, such as qPCR, to complement continued metagenomic characterization of ARGs. An understanding of these genes will allow us leverage targeted approaches that can help us better understand their mechanisms of dispersal in connected environments.

## Supporting information

S1 Fig The changes of abundance of observed ARG persister genes (average count per *recA* gene) in manure treated soil (left panel) and manure treated effluent (right panel) over time. The persister genes are on the y-axis and the same color represents the same class of antibiotic resistance. The gene abundance was averaged across 4 samples collected on the same sampling day and displayed in logarithm scale.
(TIFF)

S1 Table Metagenomes and number of ARGs associated from soil column experiment. Day is the day after manure application and represents the day of a simulated rainfall event.
(XLSX)

S2 Table ARGs identified in soil column study and their classification of origin. (XLSX)
