# Supplementary figures and images for: Investigating the dispersal of antibiotic resistance associated genes from manure application to soil and drainage waters in simulated agricultural farmland systems
Source: PLoS One. 2019 Sep 17;14(9):e0222470. doi: 10.1371/journal.pone.0222470 (PMC6748443; doi:10.1371/journal.pone.0222470)

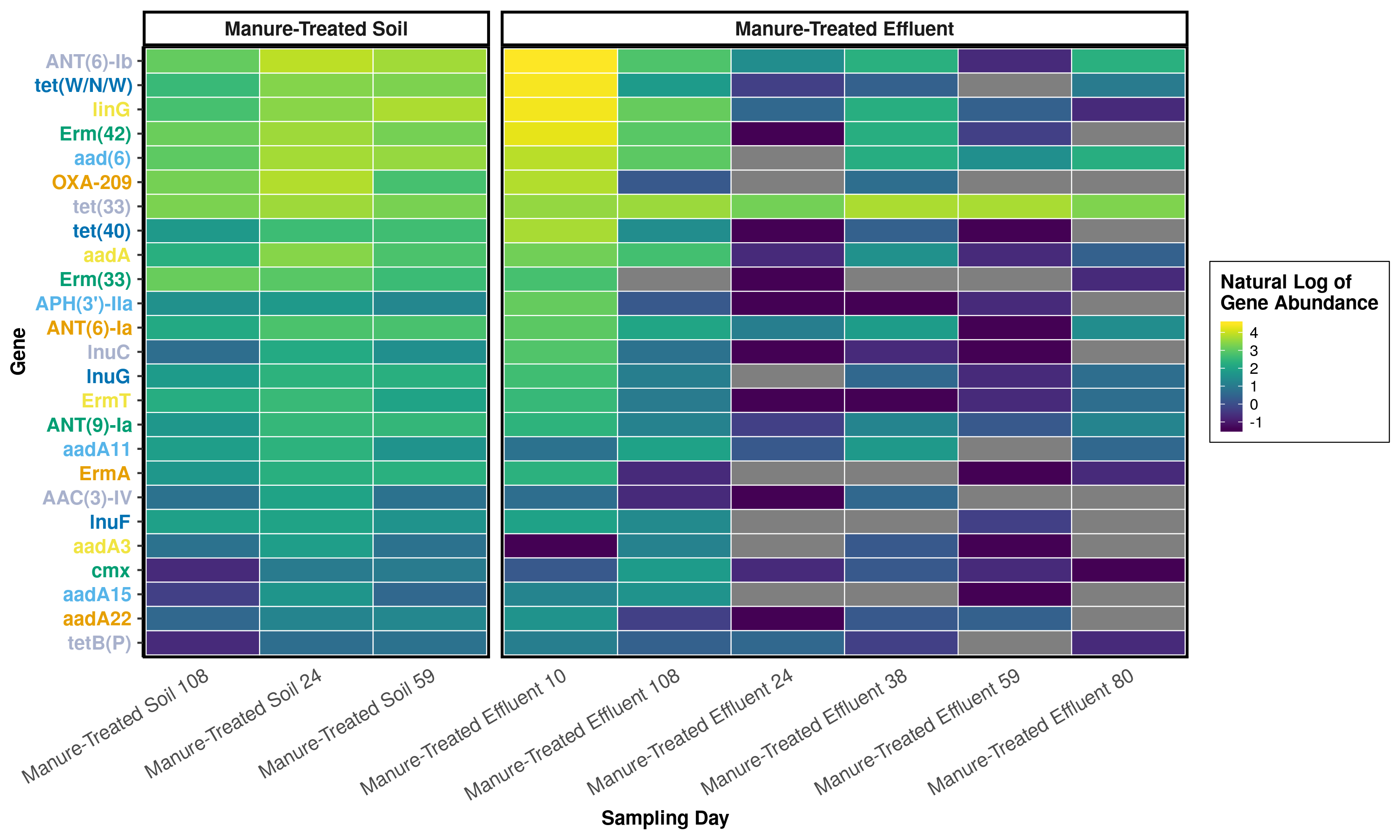

Supplement: S1 Fig — The persister genes are on the y-axis and the same color represents the same class of antibiotic resistance. The gene abundance was averaged across 4 samples collected on the same sampling day and displayed in logarithm scale. (TIFF) [file pone.0222470.s001.tiff]
